# Supplementary material for: Ubiquitous, B12-dependent virioplankton utilizing ribonucleotide-triphosphate reductase demonstrate interseasonal dynamics and associate with a diverse range of bacterial hosts in the pelagic ocean
Source: ISME Commun. 2023 Oct 3;3:108. doi: 10.1038/s43705-023-00306-9 (PMC10547690; doi:10.1038/s43705-023-00306-9)
Supplement: Supplementary file 2 — Supplementary Tables [file 43705_2023_306_MOESM2_ESM.pdf]

Supplementary Table S1. Environmental and phytoplankton abundance metadata

SampleID	Date	Day	Depth	Density(kg*m	Temperature	Salinity(PSU)	Current_head	Current vol (r	Sat_Chl(mg*r	Sat_POC(mg*	Sat_PIC(mg*r	Sat_PAR(Eins	Sat_SST (°C)	Total Autotro	Syn (Cells/mL	Proc (Cells/m	PikoEuk (Cell	NanoEuk (Cel	Virus x10^6 (	Bacteria x10^5 (Cells/mL)
May12_0m	May 12th, 20	132	0	1026.4152	16.3931936	35.983086	287.421505	0.30090738	0.22685016	66.6776831	1.2109E-05	59.9592967	17.4449997	64107.74	36756.86	4260.03	18303.03	3091.35	6900372.67	807972.39
May12_05m	May 12th, 20	132	5	1026.43446	16.4162	35.9969333	113.2	0.06062379	0.22685016	66.6776831	1.2109E-05	59.9592967	17.4449997	58490.54	33260.25	3996.13	17275.73	2818.03	6714223.6	797454.27
May12_25m	May 12th, 20	132	25	1026.5237	16.417625	35.9944167	189.175	0.04826189	0.22685016	66.6776831	1.2109E-05	59.9592967	17.4449997	52873.33	29763.63	3732.24	16248.42	2544.71	6736024.84	790102.9
May12_50m	May 12th, 20	132	50	1026.6575	16.33725	35.994625	159.375	0.06194113	0.22685016	66.6776831	1.2109E-05	59.9592967	17.4449997	49367.29	28123.71	2601.25	15739.48	2393.91	7259254.66	731518.12
May12_75m	May 12th, 20	132	75	1026.93417	15.4928333	35.9576667	199.575	0.03842525	0.22685016	66.6776831	1.2109E-05	59.9592967	17.4449997	19000.47	11309.8	1319.48	5032.86	527.79	4427888.2	450695.69
Jun8_0m	June 8th, 201	159	0	1026.22833	17.4017333	36.0534667	164.76	0.17237898	0.23117758	68.1554612	1.2887E-05	63.4369999	17.1299992	10942.35	1302.89	8042.68	871.86	440.83	1149145.78	136263.13
Jun8_05m	June 8th, 201	159	5	1026.32763	16.9490625	36.0135	62.40625	0.25205537	0.23117758	68.1554612	1.2887E-05	63.4369999	17.1299992	13185.68	1508.61	9717.83	1009.01	626.96	1337482.83	163750.44
Jun8_25m	June 8th, 201	159	25	1026.43517	16.84885	36.0052167	150.143333	0.07261246	0.23117758	68.1554612	1.2887E-05	63.4369999	17.1299992	13695.08	4124.2	6230.38	1988.63	773.9	1166820.48	156827.28
Jun8_50m	June 8th, 201	159	50	1026.68606	16.5063889	36.0846111	239.077778	0.10410502	0.23117758	68.1554612	1.2887E-05	63.4369999	17.1299992	6769.17	1430.24	3134.78	2018.02	58.78	622961.02	57761.49
Jun8_75m	June 8th, 201	159	75	1027.03215	15.6404615	36.1294615	90.3692308	0.15200257	0.23117758	68.1554612	1.2887E-05	63.4369999	17.1299992	6230.38	1459.63	2458.85	2096.39	107.76	597173.33	56382.72
Jun22_0m	June 22nd, 20	173	0	1025.90309	18.8472727	36.0994546	124.345455	0.05122903	0.21196049	64.1110166	1.7109E-05	63.5183609	19.2950001	11718.11	41.15	8004.12	2551.44	751.03	4642514.47	349484.55
Jun22_05m	June 22nd, 20	173	5	1025.95092	18.65125	36.0686667	252.9	0.13518858	0.21196049	64.1110166	1.7109E-05	63.5183609	19.2950001	11481.48	0	6965.02	2890.95	915.64	4405624.48	294567.66
Jun22_25m	June 22nd, 20	173	25	1026.4088	17.07192	36.0416	233.824	0.26230657	0.21196049	64.1110166	1.7109E-05	63.5183609	19.2950001	17860.08	72.02	12500	3312.76	1306.58	4919768.4	331474.97
Jun22_50m	June 22nd, 20	173	50	1026.73255	16.3304	36.0901	168.57	0.5715793	0.21196049	64.1110166	1.7109E-05	63.5183609	19.2950001	55102.88	39670.78	3497.94	9104.94	2150.21	4938957.82	551882.63
Jun22_75m	June 22nd, 20	173	75	1027.01775	15.4870625	36.065625	163.95625	0.38000683	0.21196049	64.1110166	1.7109E-05	63.5183609	19.2950001	4434.16	1481.48	1255.14	1121.4	92.59	3084201.82	191789.7
Jul11_0m	July 11st, 201	192	0	1025.16006	21.5934375	36.0871875	295.8375	0.23252226	0.11430367	43.7110163	0.00015111	62.6303995	20.6700001	2938.75	996.36	905.78	523.34	281.8	1468000	763873.53
Jul11_05m	July 11st, 201	192	5	1025.21167	21.4927778	36.09	298.811111	0.09218614	0.11430367	43.7110163	0.00015111	62.6303995	20.6700001	3753.95	774.94	986.29	734.69	664.24	1759111.11	856021.44
Jul11_25m	July 11st, 201	192	25	1026.00083	18.9545833	36.1210417	284.1625	0.03366411	0.11430367	43.7110163	0.00015111	62.6303995	20.6700001	3190.35	291.86	946.04	1378.8	352.25	1953333.33	942795.06
Jul11_50m	July 11st, 201	192	50	1026.66276	16.977	36.2	219.682353	0.01638465	0.11430367	43.7110163	0.00015111	62.6303995	20.6700001	7558.22	1046.68	1710.92	2254.38	1127.19	2322666.67	1096022.65
Jul11_75m	July 11st, 201	192	75	1026.90562	16.3356154	36.1741539	118.984615	0.04290101	0.11430367	43.7110163	0.00015111	62.6303995	20.6700001	8212.4	956.1	1922.26	4418.19	241.54	1739111.11	874861.64
Jul27_0m	July 27th, 201	208	0	1025.24355	21.2976364	36.0894546	61.0636364	0.07375283	0.13663873	48.4499049	0.000177	60.3527671	23.3850002	3849.15	119.22	2622.87	187.35		2551086.96	280689.78
Jul27_05m	July 27th, 201	208	5	1025.25945	21.2777273	36.0740909	44.7909091	0.20369214	0.13663873	48.4499049	0.000177	60.3527671	23.3850002	6378.35	25.55	2861.31	85.16	459.85	2169347.83	261478.1
Jul27_25m	July 27th, 201	208	25	1026.08281	18.737875	36.1536875	184.2625	0.24727257	0.13663873	48.4499049	0.000177	60.3527671	23.3850002	4164.23	17.03	2537.71	851.58	391.73	2868043.48	310759.12
Jul27_50m	July 27th, 201	208	50	1026.56178	17.1333333	36.1173333	132.055556	0.18117153	0.13663873	48.4499049	0.000177	60.3527671	23.3850002	13046.23	2946.47	3593.67	5279.81	630.17	2538695.65	297372.26
Jul27_75m	July 27th, 201	208	75	1026.979	15.85175	36.12275	307.925	0.08543276	0.13663873	48.4499049	0.000177	60.3527671	23.3850002	10278.59	1388.08	5194.65	3287.1	187.35	1636521.74	120941.61
Aug5_0m	August 5th, 2	217	0	1025.06785	21.9334615	36.0909231	313.584615	0.22378113	0.14524373	51.1999054	0.000182	58.6197134	23.0949993	17666.89	6832.56	1568.87	7617	989.07	1047399.96	412682.12
Aug5_05m	August 5th, 2	217	5	1025.13681	21.8215	36.112125	71.49375	0.20292673	0.14524373	51.1999054	0.000182	58.6197134	23.0949993	24738.19	9583.77	1898.57	11232.23	1261.92	1245522.98	1049406.29
Aug5_25m	August 5th, 2	217	25	1026.04113	18.6854667	36.0836	54.82	0.03887101	0.14524373	51.1999054	0.000182	58.6197134	23.0949993	26398.01	12016.67	1978.15	10231.79	1625.72	1414941.56	601867.88
Aug5_50m	August 5th, 2	217	50	1026.59569	16.901625	36.089	235.675	0.05768753	0.14524373	51.1999054	0.000182	58.6197134	23.0949993	33196.47	16723.29	6468.76	8594.7	886.75	1565639.86	673695.03
Aug5_75m	August 5th, 2	217	75	1026.94953	15.8157059	36.0729412	173.464706	0.23643618	0.14524373	51.1999054	0.000182	58.6197134	23.0949993	14369.98	3717.55	5491.06	3421.96	454.75	807780.31	160741.39
Sep8_0m*	September 8t	251	0	1024.965	21.80276	35.872982	NA	0.08259867	0.11669481	42.8443498	1.27E-05	48.5509349	22.8850002	9730.79	6288.42	704.74	1825.09	722.81	3557722.93	252401.5
Sep8_05m*	September 8t	251	5	1024.99	21.709738	35.871456	NA	0.10939939	0.11669481	42.8443498	1.27E-05	48.5509349	22.8850002	9685.61	6180	614.39	1797.98	731.84	3603403.66	285722
Sep8_25m*	September 8t	251	25	1025.333	20.454329	35.86993	NA	0.14939488	0.11669481	42.8443498	1.27E-05	48.5509349	22.8850002	9007.98	4987.37	876.4	1969.65	650.53	3384136.15	313326
Sep8_50m*	September 8t	251	50	1025.95	17.96768	35.837887	NA	0.12136702	0.11669481	42.8443498	1.27E-05	48.5509349	22.8850002	15892.72	4589.82	2132.28	5899.91	1165.53	5558807.72	509695.5
Sep8_75m*	September 8t	251	75	1026.531	15.499344	35.83331	NA	0.10140599	0.11669481	42.8443498	1.27E-05	48.5509349	22.8850002	9450.7	1852.19	3532.72	3523.68	252.98	3650965.37	383778

*Equipment malfunction prevented CTD data collection on September 8, thus, temperature, salinity, and density metadata was estimated from the nearest time, pixel, and depth from the Marine Copernicus Global Ocean Physics model

Supplementary Table S2. Determination of environmental factors correlated with community composition

(A) Mantel test between virioplankton composition similarity matrix based on Class II RTPR phylogenetic clades and metadata similarity matrices

Tested metadata	Spearman		Pearson	
	r	p-value	r	p-value
Day	0.42	0.001*	0.42	0.001*
Depth	0.19	0.008*	0.22	0.006*
Temperature	0.48	0.001*	0.51	0.001*
Density	0.44	0.001*	0.48	0.001*
Current Velocity	0.07	0.211	-0.01	0.496
Salinity	0.16	0.029*	0.12	0.091
Nanoeukaryote Abundance	0.06	0.226	0.02	0.376
Picoeukaryote Abundance	0.10	0.085	0.05	0.256
Prochlorococcus Abundance	-0.08	0.861	-0.09	0.877
Synechococcus Abundance	0.12	0.096	0.02	0.398
Total Autotroph Abundance	0.05	0.276	0.01	0.413
Viral abundance	0.03	0.315	0.04	0.297
Bacterial abundance	-0.08	0.911	-0.10	0.951

(B) Mantel test between virioplankton composition similarity matrix based on Class II RTPR 98% OTUs and metadata similarity matrices

Tested metadata	Spearman		Pearson	
	r	p-value	r	p-value
Day	0.30	0.001*	0.27	0.001*
Depth	0.15	0.014*	0.17	0.005*
Temperature	0.38	0.001*	0.44	0.001*
Density	0.35	0.001*	0.43	0.001*
Current Velocity	0.11	0.143	0.06	0.289
Salinity	0.14	0.081	0.09	0.197
Nanoeukaryote	0.14	0.065	0.09	0.197
Picoeukaryote Abundance	0.14	0.079	0.07	0.216
Prochlorococcus	-0.13	0.938	-0.16	0.967
Synechococcus Abundance	0.09	0.159	0.02	0.387
Total Autotroph	0.09	0.154	0.02	0.387
Viral abundance	0.08	0.152	0.08	0.194
Bacterial abundance	-0.03	0.627	-0.04	0.694

(C) Mantel test between bacterioplankton composition similarity matrix and metadata similarity matrices

Tested metadata	Spearman		Pearson	
	r	p-value	r	p-value
Day	0.35	0.001*	0.37	0.001*
Depth	0.45	0.001*	0.47	0.001*
Temperature	0.42	0.001*	0.46	0.001*
Density	0.46	0.001*	0.50	0.001*
Current Velocity	-0.04	0.690	-0.02	0.878
Salinity	0.15	0.103	0.15	0.145
Nanoeukaryote Abundance	0.10	0.326	0.08	0.493
Picoeukaryote Abundance	0.06	0.549	0.04	0.733
Prochlorococcus Abundance	-0.01	0.945	-0.08	0.493
Synechococcus Abundance	0.11	0.282	0.03	0.745
Total Autotroph Abundance	0.08	0.441	0.05	0.642
Viral abundance	0.11	0.122	0.10	0.248
Bacterial abundance	0.06	0.432	0.04	0.643

**p* -value <.05 permutation = 999

Supplementary Table S3. Beta group diversity significance based on day and depth

(A) PERMANOVA test of virioplankton community beta group diversity significance based on Class II RTPR phylogenetic clades across sampling day					
Group 1	Group2	Sample size	pseudo-F	p-value	q-value
12-May	8-Jun	7	1.272	0.334	0.438
12-May	22-Jun	8	4.340	0.024*	0.091
12-May	Jul11	9	5.096	0.019*	0.091
12-May	Jul27	8	9.294	0.026*	0.091
12-May	Aug5	9	15.921	0.012*	0.091
12-May	Sep8	8	3.191	0.025*	0.091
8-Jun	Jun22	7	1.950	0.097	0.185
8-Jun	Jul11	8	2.453	0.052	0.109
8-Jun	Jul27	7	4.299	0.032*	0.096
8-Jun	Aug5	8	8.345	0.022*	0.091
8-Jun	Sep8	7	1.586	0.261	0.365
22-Jun	Jul11	9	0.574	0.750	0.788
22-Jun	27-Jul	8	1.398	0.246	0.365
22-Jun	Aug5	9	3.985	0.037*	0.097
22-Jun	Sep8	8	0.558	0.823	0.823
11-Jul	Jul27	9	1.030	0.428	0.529
11-Jul	Aug5	10	3.476	0.042*	0.098
11-Jul	Sep8	9	0.638	0.728	0.788
27-Jul	Aug5	9	1.777	0.141	0.228
27-Jul	Sep8	8	0.830	0.555	0.648
5-Aug	Sep8	9	1.610	0.133	0.228

(B) PERMANOVA test of virioplankton community beta group diversity significance based on Class II RTPR 98% OTUs across sampling day					
Group 1	Group2	Sample size	pseudo-F	p-value	q-value
12-May	8-Jun	7	1.827	0.04*	0.095
12-May	22-Jun	8	1.263	0.153	0.262
12-May	Jul11	9	2.101	0.016*	0.090
12-May	Jul27	8	2.437	0.025*	0.090
12-May	Aug5	9	3.749	0.008*	0.090
12-May	Sep8	8	1.983	0.03*	0.090
8-Jun	Jun22	7	1.365	0.140	0.262
8-Jun	Jul11	8	1.611	0.045*	0.105
8-Jun	Jul27	7	1.989	0.056	0.118
8-Jun	Aug5	8	3.882	0.019*	0.090
8-Jun	Sep8	7	1.458	0.176	0.264
22-Jun	Jul11	9	0.890	0.640	0.704
22-Jun	27-Jul	8	1.103	0.256	0.358
22-Jun	Aug5	9	2.019	0.026*	0.090
22-Jun	Sep8	8	0.915	0.505	0.589
11-Jul	Jul27	9	1.017	0.369	0.456
11-Jul	Aug5	10	2.164	0.029*	0.090
11-Jul	Sep8	9	0.869	0.670	0.704
27-Jul	Aug5	9	1.066	0.281	0.369
27-Jul	Sep8	8	0.761	0.770	0.770
5-Aug	Sep8	9	1.480	0.162	0.262

(C) PERMANOVA test of bacterioplankton community beta group diversity significance across sampling day					
Group 1	Group2	Sample size	pseudo-F	p-value	q-value
12-May	8-Jun	10	2.066	0.005*	0.042*
12-May	22-Jun	10	2.229	0.023*	0.069
12-May	Jul11	10	3.922	0.007*	0.042*
12-May	Jul27	10	3.548	0.009*	0.042*
12-May	Aug5	10	3.141	0.012*	0.042*
12-May	Sep8	10	4.365	0.008*	0.042*
8-Jun	Jun22	10	1.147	0.263	0.29
8-Jun	Jul11	10	2.371	0.036*	0.084
8-Jun	Jul27	10	1.891	0.042*	0.088
8-Jun	Aug5	10	2.046	0.031*	0.081
8-Jun	Sep8	10	2.988	0.012*	0.042*
22-Jun	Jul11	10	1.586	0.136	0.196
22-Jun	27-Jul	10	1.328	0.169	0.209
22-Jun	Aug5	10	1.449	0.147	0.196
22-Jun	Sep8	10	2.291	0.058	0.111
11-Jul	Jul27	10	1.13	0.277	0.291
11-Jul	Aug5	10	1.191	0.236	0.275
11-Jul	Sep8	10	1.548	0.113	0.196
27-Jul	Aug5	10	0.929	0.464	0.464
27-Jul	Sep8	10	1.604	0.131	0.196
5-Aug	Sep8	10	1.486	0.149	0.196

[illegible] significance based on Class II RTPR phylogenetic clades across sampling depths					
Group 1	Group2	Sample size	pseudo-F	p-value	q-value
0m	5m	10	2.268	0.101	0.165
0m	25m	10	2.026	0.115	0.165
0m	50m	11	3.105	0.039*	0.125
0m	75m	10	6.688	0.01*	0.100
5m	25m	12	1.318	0.237	0.263
5m	50m	13	2.503	0.050	0.125
5m	75m	12	3.492	0.025*	0.125
25m	50m	13	0.557	0.685	0.685
25m	75m	12	1.760	0.121	0.165
50m	75m	13	1.769	0.132[illegible]	0.165

(D) PERMANOVA test of virioplankton community beta group diversity significance based on Class II RTPR 98% OTUs across sampling depths					
Group 1	Group2	Sample size	pseudo-F	p-value	q-value
0m	5m	10	1.104	0.294	0.368
0m	25m	10	1.246	0.217	0.362
0m	50m	11	1.608	0.067	0.223
0m	75m	10	2.370	0.013*	0.130
5m	25m	12	0.944	0.458	0.509
5m	50m	13	1.125	0.279	0.368
5m	75m	12	0.869	0.571	0.571
25m	50m	13	1.255	0.146	0.362
25m	75m	12	1.738	0.055	0.223
50m	75m	13	1.242	0.183	0.362

(F) PERMANOVA test of bacterioplankton community beta group diversity significance across sampling depths					
Group 1	Group2	Sample size	pseudo-F	p-value	q-value
0m	5m	14	0.412	0.993	0.993
0m	25m	14	0.862	0.561	0.701
0m	50m	14	2.318	0.013*	0.026*
0m	75m	14	4.469	0.001*	0.001*
5m	25m	14	0.847	0.634	0.704
5m	50m	14	2.388	0.007*	0.018*
5m	75m	14	4.596	0.002*	0.007*
25m	50m	14	1.22	0.171	0.244
25m	75m	14	3.183	0.002*	0.007*
50m	75m	14	1.519	0.040*	0.067

Permutation time: 999; three decimal places were kept.

*p-value or q-value < 0.05
